# Discovery of Microorganisms and Enzymes Involved in High-Solids Decomposition of Rice Straw Using Metagenomic Analyses

**DOI:** 10.1371/journal.pone.0077985

**Published:** 2013-10-25

**Authors:** Amitha P. Reddy, Christopher W. Simmons, Patrik D’haeseleer, Jane Khudyakov, Helcio Burd, Masood Hadi, Blake A. Simmons, Steven W. Singer, Michael P. Thelen, Jean S. VanderGheynst

**Affiliations:** 1 Joint BioEnergy Institute, Emeryville, California, United States of America; 2 Biological and Agricultural Engineering, University of California Davis, Davis, California, United States of America; 3 Food Science, University of California Davis, Davis, California, United States of America; 4 Earth Sciences Division, Lawrence Berkeley National Laboratory, Berkeley, California, United States of America; 5 Physical and Life Sciences Directorate, Lawrence Livermore National Laboratory, Livermore, California, United States of America; 6 Biological and Materials Science Center, Sandia National Laboratories, Livermore, California, United States of America; 7 Physical Biosciences Division, Lawrence Berkeley National Laboratory, Berkeley, California, United States of America; Auburn University, United States of America

## Abstract

High-solids incubations were performed to enrich for microbial communities and enzymes that decompose rice straw under mesophilic (35°C) and thermophilic (55°C) conditions. Thermophilic enrichments yielded a community that was 7.5 times more metabolically active on rice straw than mesophilic enrichments. Extracted xylanase and endoglucanse activities were also 2.6 and 13.4 times greater, respectively, for thermophilic enrichments. Metagenome sequencing was performed on enriched communities to determine community composition and mine for genes encoding lignocellulolytic enzymes. *Proteobacteria* were found to dominate the mesophilic community while *Actinobacteria* were most abundant in the thermophilic community. Analysis of protein family representation in each metagenome indicated that cellobiohydrolases containing carbohydrate binding module 2 (CBM2) were significantly overrepresented in the thermophilic community. *Micromonospora*, a member of *Actinobacteria*, primarily housed these genes in the thermophilic community. In light of these findings, *Micromonospora* and other closely related *Actinobacteria* genera appear to be promising sources of thermophilic lignocellulolytic enzymes for rice straw deconstruction under high-solids conditions. Furthermore, these discoveries warrant future research to determine if exoglucanases with CBM2 represent thermostable enzymes tolerant to the process conditions expected to be encountered during industrial biofuel production.

## Introduction

Considerable efforts are underway to identify plant sources and conversion technologies to enable economical and sustainable production of fuels and chemicals from plant biomass and meet renewable fuel standards [1]–[4]. Agricultural residues are a promising resource because they do not compete with land used for food production [5]–[8]. Residues of particular interest are the hulls and straw associated with rice cultivation, harvest and processing. In 2010 worldwide rice production exceeded 690 million tons on 159 million ha of land [9] with estimated rice straw generation of 5.6–6.7 t/ha (890–1,065 million dry tons in 2010) [5], [10], [11]. While rice straw could be a significant resource for biofuel feedstock, challenges related to pretreatment and enzymatic hydrolysis have prevented its widespread conversion to biofuel. The development of cost-effective enzymes that efficiently hydrolyze plant cell wall polysaccharides under industrially relevant conditions would enable biofuel production from plant biomass feedstocks like rice straw [12], [13].

Microbial communities that decompose plant cell wall polymers (lignocellulose) in extreme environments have been identified as a promising source of hydrolyzing enzymes [14], [15]. Discovery of enzymes in these types of environments is particularly challenging due to a number of factors, including the tendency of carbohydrate-active enzymes to bind to substrates and interference by compounds present in lignocellulosic biomass when analyzing proteins and other metabolites. Approaches based on nucleic acid analyses offer alternatives that may overcome traditional methods of microorganism and enzyme discovery [16], [17].

The goal of this research was to use a combination of enrichment and metagenomic approaches to discover promising organisms and enzymes for the efficient hydrolysis of rice straw. Recognizing that bioconversion processes may occur over a range of temperatures and in high-solids environments, enrichments were completed as solid fermentations at 35°C and 55°C.

## Materials and Methods

### High Solids Incubations

Finished green waste compost was obtained from a commercial facility that composts agricultural residues including tree and vine prunings, with permission from Greg Kelly (Northern Recycling, Zamora, CA). Compost was solar-dried and stored at 4°C until applied as inocula. Fresh rice straw (*Oryza sativa L.*, California rice M206) was collected as described previously [18]. The dried straw was extracted with ethanol for 1.5 days and water for 2 days in a soxhlet extractor, dried in a vacuum oven for 4 days to 3.2% moisture on a dry basis (3.1% on a wet basis), and stored in zipper lock bags at 4°C until needed.

High-solids incubations were conducted as described previously with minor modifications [15]. Briefly, bioreactors with a 0.2 L working volume were loaded with 5–10 g dry weight of rice straw and inocula mixture. Prior to incubation, rice straw was wetted with minimal media [19] to a moisture content of 400 wt% dry basis (g water g dry solid^−1^) and equilibrated at 4°C overnight. For the initial enrichment in each experiment, wetted rice straw was inoculated with 10 wt% (g dry compost (g dry solid)^−1^) compost. Every 6 to 7 days, fresh feedstock was inoculated with 10 wt% (g dry enriched sample (g total dry weight)^−1^) of the enriched community and transferred to a new bioreactor.

Incubator temperature was maintained at 35°C for mesophilic incubations. For the first enrichment of thermophilic incubations, the incubator temperature was maintained at 35°C for 1 day, ramped to 55°C over one day, and held at 55°C for the duration of the experiment. Water lost during incubation was replaced and each bioreactor was mixed every 3.5 days.

Microbial community respiration rate, represented as CO_2_ evolution rate (CER), was measured for all incubated samples. Carbon dioxide concentration was measured on the influent and effluent air of the bioreactors using an infrared CO_2_ sensor (Vaisala, Woburn, MA) and flow was measured with a thermal mass flow meter (Aalborg, Orangeburg, NY). Carbon dioxide and flow data were recorded every 20 min using a data acquisition system. Carbon dioxide evolution rate and cumulative respiration (cCER) were calculated as described previously [20].

Two sets of enrichments were completed. Enrichments for selection of an enzyme extraction buffer ran for 5 weeks. The second set of enrichments ran for four weeks, yielding a total of four sampling points (T1, T2, T3 and T4) for enzyme activity measurement. The T4 sampling point consisted of three replicate enrichments while T1, T2 and T3 where individual enrichments. Samples from the T4 sampling point were collected for DNA extraction.

### Enzyme Extraction from Solid Samples

Buffer components for enzyme extraction were selected using a full factorial experiment (Table 1). Extractions were conducted with ethylene glycol (0–50 wt%), Tween 80 (0.01–0.15 wt %) and NaCl (0.1–1.5 wt %) and a sodium acetate buffer (50 mM, pH = 5.0) control.

**Table 1:** Experimental design for enzyme extraction from rice straw and corresponding enzyme activities.

	Coded Design Setting			Xylanase	Endogucanase
Treatment	NaCl*	Tween 80**	Ethylene Glycol***	(IU gdw^−1^)	(IU gdw^−1^)
1	0	0	0	0.81	0.21
2	+1	−1	−1	0.76	0.21
3	0	0	0	0.70	0.19
4	+1	+1	+1	1.27	0.23
5	−1	−1	−1	0.60	0.22
6	−1	+1	+1	1.25	0.34
7	+1	−1	+1	1.08	0.23
8	−1	−1	+1	0.88	0.22
9	+1	+1	−1	0.83	0.16
10	0	0	0	0.77	0.18
11	−1	+1	−1	0.71	0.12
12	Sodium Acetate buffer control			0.85	0.19

* NaCl: −1 = 0.1 wt%, 0 = 0.8 wt%, +1 = 1.5 wt%.

** Tween 80: −1 = 0.01 wt%, 0 = 0.08 wt%, +1 = 0.15 wt%.

*** Ethylene Glycol: −1 = 0 wt%, 0 = 25 wt%, +1 = 50 wt%.

To extract enzymes, three grams (wet weight) of freshly harvested colonized feedstock was shaken with 27 g of buffer for 60 minutes at 150 RPM and room temperature. Samples were centrifuged at 4°C and 10,000×g for 20 min and then vacuum filtered using 0.2 µm membranes. The extraction buffer was exchanged with sodium acetate buffer using VivaSpin columns with a PES membrane and a 5 kDa molecular weight cut off (VWR, West Chester, PA). Endoglucanase and xylanase activities in dialyzed extracts were measured as described previously [15]. JMP statistical software (v. 8.0.1, SAS Institute, Cary, NC) was used to perform stepwise regression and determine significant buffer components.

For samples T2–T4, enzymes were extracted using 50 wt% ethylene glycol, 0.15 wt% Tween 80 and 1.5 wt% NaCl and assayed according to methods described elsewhere [15]. All assays were completed in triplicate. Activities were reported as IU gdw^−1^ where one IU = µmol product min^−1^.

### DNA Extraction

Samples from the T4 time point were frozen in liquid nitrogen, homogenized with an oscillating ball mill (MM400, Retsch Inc., Newtown, PA), and stored with LifeGuard Soil Preservation Solution (Mo Bio Laboratories, Inc., Carlsbad, CA) in a ratio of 1∶2.5 (sample:LifeGuard) at −80°C. Samples were thawed on ice and processed with the MoBio PowerSoil DNA Isolation kit (Mo Bio Laboratories, Inc., Carlsbad, CA).

### 16S rDNA Library Construction, Sequencing, and Binning

A fragment of the 16S small-subunit rRNA gene was PCR-amplified from DNA extracts using the primer sequences 926F and 1392R containing 454 adapters and barcodes using a previously described method [21]. AMPure Solid Phase Reversible Immobilization (SPRI) beads (Beckman Coulter) were used to purify amplicons. Emulsion PCR was performed using a GS FLX Titanium MV emPCR Kit (Roche). A Genome Sequencer FLX instrument and associated Titanium series kits (Roche) were used for sequencing of amplicons. Sequencing reads were analyzed using the methods of Kunin et al. [22]. In brief, PyroTagger software (Joint Genome Institute) was used to quality trim base calls, trim primer sequences from reads, remove duplicate reads, and bin reads by performing blastn alignments against the Greengenes database using default settings [23].

### Metagenome Sequencing, Assembly, and Annotation

DNA fragments for 454 and Illumina sequencing were created using the Joint Genome Institute standard library generation protocols for Roche 454 GS FLX Titanium and Illumina HiSeq 2000 platforms. Metagenome sequencing was performed using a Roche GS FLX Titanium sequencing kit on a Roche/454 FLX-Ti system. Illumina sequencing was performed on a HiSeq 2000 system. Combined sequencing reads from 454 and Illumina runs were quality trimmed using a quality threshold of 10. Trimmed reads were assembled with SOAPdenovo [24] and Newbler [25] for contigs >1800 bp. A minimum overlap identity of 98% and a minimum overlap length of 80 bases were used. Contigs longer than 1800 bp and contigs resulting from Newbler assembly were assembled into a single assembly using Minimus [26] with a minimum overlap length of 80 bases, a minimum overlap identity of 98%, and a consensus error of 0.06 for joining. Burrows-Wheeler Aligner [27] was used to map reads back to contigs in order to confirm proper placement and calculate read depth for contigs. Annotation of contigs was performed using the Joint Genome Institute’s Integrated Microbial Genomes with Microbiomes-Expert Review (IMG/M-ER) pipeline [28].

### Contig Binning

All contigs were scanned for genes within phylogenetic marker COGs using the IMG/M toolset [29]. The IMG/M pre-set list of marker COGs was used. Amino acid sequences of detected marker COG genes were imported into the Galaxy platform [30]–[32]. The blastp function of Galaxy was used to align marker COG genes against the NCBI protein database with an E-value cutoff of 1e-10. The best blast hit for each marker COG gene was used to bin its contig of origin at the genus level. For contigs with more than one marker COG gene, the taxonomy for over 50% of marker COG genes had to agree for the contig to be binned. Contigs with marker COG genes stemming from the same genus were collated into binning training sets. ClaMS software was used for supervised binning of metagenome contigs seeded with genus training sets [33]. Within ClaMS, De Bruijn chain signatures were used as the metric for binning with a kmer length of 2 and a signature cutoff value of 0.005.

### Metagenome Analysis

R software running the VEGAN package [34] was used to determine the Shannon index, richness, and Pielou index of each community based on pyrotag data. Rarefaction curves were generated from pyrotag data using PAST software [35]. Similarity percentage (SIMPER) analysis was executed as described previously [36]. IMG/M was used for comparative genomics. The abundance profile search tool was used to find differences in protein family representation between the two metagenomes. Protein families from the Pfam database were used [37]. For the search, gene counts were normalized by the total number of genes in a given metagenome. Gene counts refer to the number of homologs in a metagenome for a given gene and do not factor in gene copy number. As a result, gene counts indicate how many different versions of a particular gene exist within a metagenome and are not skewed by how abundant the source microorganisms are in the community. Search criteria were set to find only protein families for which normalized gene counts were at least twice as abundant in the thermophilic enrichment community compared to the mesophilic enrichment community. Gene counts between communities were compared using the D-score statistic [29] with a minimum gene count threshold of 5. A false discovery rate of 0.05 was used for determining statistical significance.

For select protein families identified through the abundance search, genes were analyzed with respect to their clusters of orthologous genes (COG) group. Genes from deconstruction-relevant COGs were aligned using MUSCLE [38] and processed in Phylip [39] to perform bootstrapping with 1000 replicates, generate F84 distance matrices, and perform neighbor-joining. Phylip was used to find the consensus tree using a majority rule to retain branches present in ≥50% of bootstrap replicates. The cellobiohydrolase CelD gene from *Aspergillus fumigatus* was used as an outgroup.

### Data Archiving

Metagenome raw reads, assembled scaffolds, and gene annotations can be accessed through IMG/M. The metagenomes are listed as Taxon Object ID 2199352012 (Mesophilic rice straw/compost enrichment metagenome: eDNA_1 (Mesophilic 454/Illumina Combined June 2011 assem)) and Taxon Object ID 2199352008 (Thermophilic rice straw/compost enrichment metagenome: eDNA_2 (Thermophilic 454/Illumina Combined June 2011 assem)).

## Results

### Identification of Extraction Buffer

The enzyme activities extracted from incubated rice straw are presented in Table 1. Xylanase activities from rice straw varied between 0.85 IU g dw^−1^ for sodium acetate extraction to 1.25–1.27 IU (g dw)^−1^ for extractions containing 50 wt% ethylene glycol and 0.15 wt% Tween 80 in the presence of either 0.1 wt% NaCl or 1.5 wt% NaCl. Endoglucanase extraction also varied with the composition of the buffer, but differences were much smaller compared to xylanase. Like xylanase, the highest activity, 0.34 IU (g dw)^−1^ was observed with extractions containing 50 wt% ethylene glycol and 0.15 wt% Tween 80.

Ethylene glycol had a significant positive effect on xylanase (p<0.001) and endoglucanase (p-value<0.02) extractions. For both xylanase and endoglucanase extraction, the interaction between Tween 80 and ethylene glycol was significant. When ethylene glycol was at 50 wt% in the buffer, increasing Tween 80 from 0.01 wt% to 0.15 wt% increased xylanase extraction (p-value = 0.036) and endoglucanase extraction (p-value = 0.029). Sodium chloride had a significant positive effect on xylanase activity extracted from rice straw (p-value = 0.021), but had no effect on endoglucanase activity (p-value>0.05).

### Temperature Effects on Microbial Activity and Extracted Endoglucanase and Xylanase Activities

Microbial respiration and extracted enzymatic activity were greater for thermophilic compared to mesophilic incubations (Table 2). For the T4 sampling point, cumulative respiration was 7.5 times greater at 55°C compared to 35°C, while extracted xylanase and endoglucanase activities were 2.6 and 13.4 times greater, respectively. For 35°C incubations, there was little change in cumulative respiration and extracted enzyme activities with enrichment. In contrast, respiration increased by a factor of 3 between enrichments T2 and T4 at 55°C. Similar changes were observed in the activity of extracted enzymes. Xylanase and endoglucanase activity increased by factors of 2.7 and 1 between enrichments T2 and T4, respectively.

**Table 2:** Cumulative carbon dioxide evolution rate after 7 days of incubation (cCER) and extracted enzyme activity after each incubation period.

	cCER		Xylanase*		Endoglucanase*		
	(mg CO_2_ (g dry feedstock)^−1^)		(IU gdw^−1^)		(IU gdw^−1^)		
Sampling point	35°C	55°C	35°C	55°C	35°C	55°C	
T1	35	109	**	**	**	**	
T2	36	95	3.82 (0.06)	2.0 (0.07)	0.70 (0.03)	0.68 (0.03)	
T3	48	246	2.53 (0.05)	11.5 (0.4)	0.40 (0.06)	1.9 (0.3)	
T4***	39 (4)	292 (19)	2.07 (0.04)	7.4 (0.1)	0.094 (0.007)	1.35 (0.02)	

* Values for xylanase and endoglucanase activities are given as means for triplicate assays with one standard deviation given in parentheses.

** data not available.

*** n = 3 for T4 respiration measurements. Values in parentheses represent one standard deviation.

### Metagenome Sequencing and Assembly

Illumina and 454 sequencing of mesophilic and thermophilic communities yielded total read counts of 447,683,681 and 448,669,837, respectively. Of these reads, 94.3% of reads from the mesophilic community passed quality filtering, while 91.1% of thermophilic community reads passed. Assembly of filtered reads resulted in 264,109 contigs for the mesophilic community and 512,311 contigs for the thermophilic community.

### Microbial Community Composition

**Figure 1:**
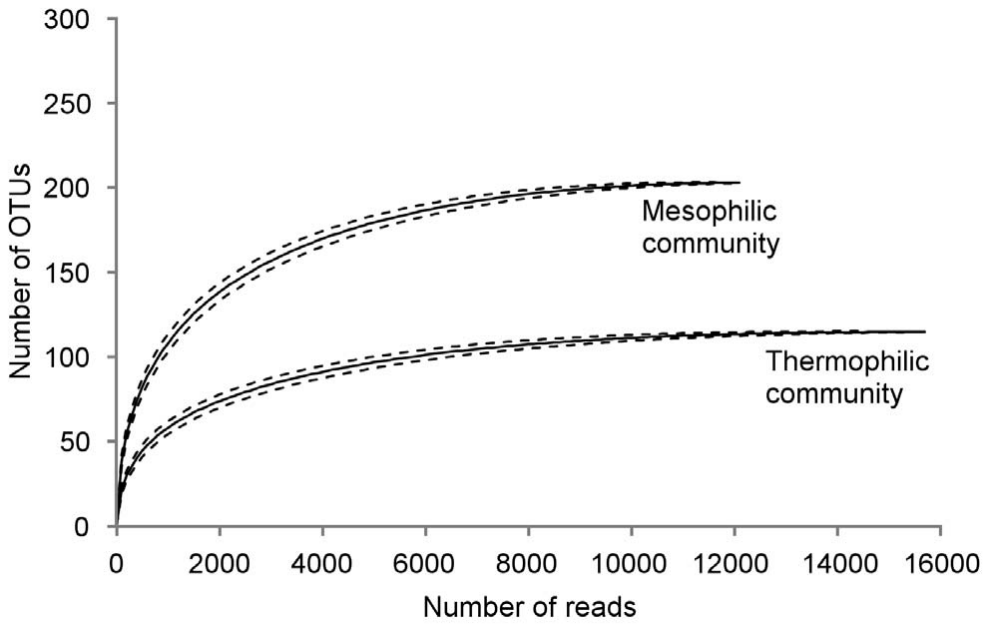
Rarefaction curves from pyrotag data for enriched mesophilic and thermophilic microbial communities. Dashed lines indicate ±1 standard error.

**Figure 2:**
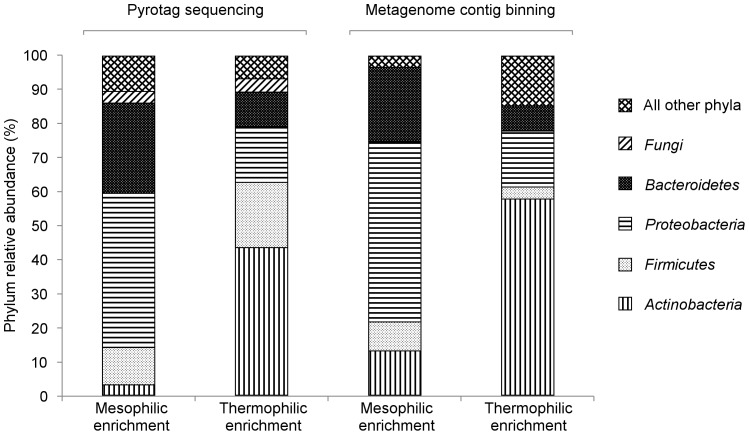
Pylum composition of microbial communities from mesophilic and thermophilic enrichments on rice straw.

Rarefaction curves generated from pyrotag reads showed a clear asymptote for both communities, indicating sufficient sampling to capture most operational taxonomic units (OTUs) within communities (Figure 1). Pyrotag sequencing revealed that microbial communities from the thermophilic enrichment were less diverse than those from the mesophilic enrichment (Table 3). Decreased diversity in the thermophilic community, as indicated by a lower Shannon index relative to the mesophilic community, stemmed from decreased richness and evenness during thermophilic enrichment. Differences in microbial community structure between mesophilic and thermophilic enrichments were primarily a result of differences in abundance for *Actinobacteria*, *Firmicutes*, *Proteobacteria*, and *Bacteroidetes* bacteria (Figure 2). Both pyrotag sequencing and abundance data for metagenome contigs containing 16S rRNA genes indicated enrichment of *Actinobacteria* under thermophilic conditions relative to mesophilic conditions. Alternately, the data showed decreases in relative abundance for *Proteobacteria* and *Bacteroidetes* in the thermophilic culture compared to the mesophilic culture. SIMPER analysis of binned metagenome contigs revealed that genera within *Actinobacteria* were the largest contributors to dissimilarity between the thermophilic and mesophilic communities (Table 4). Increased abundance of *Micromonospora* and *Mycobacterium* in the thermophilic community accounted for approximately one third of the Bray-Curtis dissimilarity between the two communities. Decreased abundance of *Chryseobacterium* and *Pseudoxanthomonas* (members of *Bacteroidetes* and *Proteobacteria*, respectively) in the thermophilic community was also a major contributor to the overall dissimilarity between the thermophilic and mesophilic communities.

**Table 3:** Ecological measures for microbial communities from mesophilic and thermophilic enrichments on rice straw.

Method	Enrichment	Shannon index, H	Richness, S	Pielou index, J
Pyrotag sequencing	Mesophilic	3.62	204	0.68
	Thermophilic	2.49	115	0.52

**Table 4:** SIMPER analysis of genera accounting for >75% of dissimilarity between thermophilic and mesophilic microbial communities based on metagenome binning.

Taxonomy	Thermophilic enrichment relative abundance	Mesophilic enrichment relative abundance	% contribution
*Micromonospora* (*Actinobacteria*)	30.5	0.0	19.3
*Mycobacterium* (*Actinobacteria*)	20.4	0.0	12.9
*Chryseobacterium* (*Bacteroidetes*)	0.0	14.7	9.3
*Pseudoxanthomonas* (*Proteobacteria*)	11.6	24.3	8.1
*Conexibacter* (*Actinobacteria*)	0.0	9.9	6.3
*Phenylobacterium* (*Proteobacteria*)	0.0	8.6	5.5
*Thermobifida* (*Actinobacteria*)	6.8	0.0	4.3
*Brevundimonas* (*Proteobacteria*)	0.0	6.4	4.1
*Candidatus Solibacter* (*Acidobacteria*)	5.5	0.0	3.5
*Brevibacillus* (*Firmicutes*)	0.0	4.3	2.7

**Figure 3:**
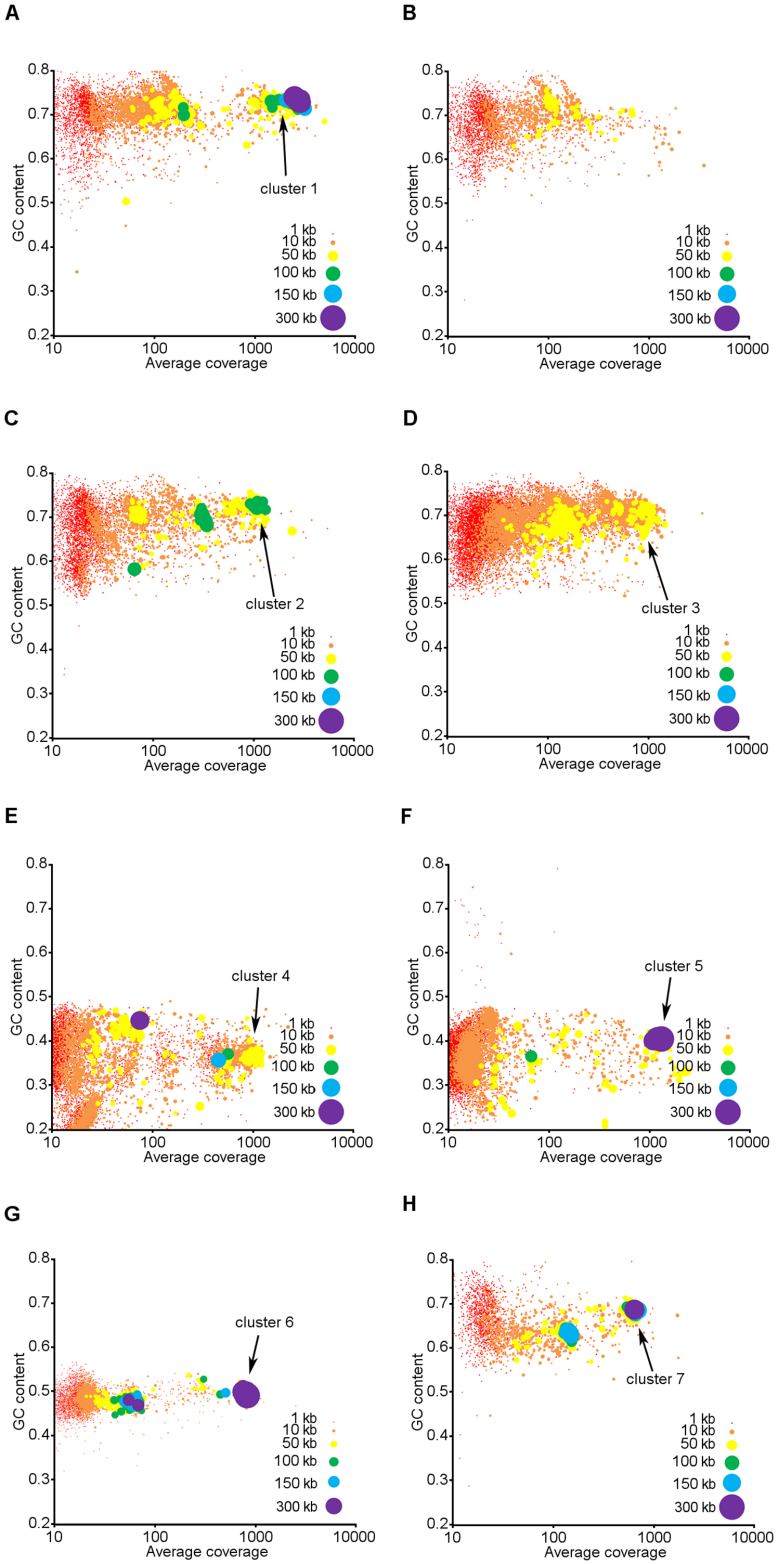
Scatterplots of contig properties for select genus bins in thermophilic and mesophilic communities. Plotted contigs correspond to (A) *Micromonospora* (*Actinobacteria*) in thermophilic community, (B) *Mycobacterium* (*Actinobacteria*) in thermophilic community, (C) *Pseudoxanthomonas* (*Proteobacteria*) in thermophilic community, (D) *Pseudoxanthomonas* (*Proteobacteria*) in mesophilic community, (E) *Chryseobacterium* (*Bacteroidetes*) in mesophilic community, (F) *Niabella* (*Bacteroidetes*) in thermophilic community, (G) *Niastella* (*Bacteroidetes*) in mesophilic community, and (H) *Chelativorans* (*Proteobacteria*) in thermophilic community. Genera presented in A–E account for >50% of total dissimilarity between thermophilic and mesophilic communities. Notable clusters with high abundance or large contigs are labeled for reference in subsequent analyses.

Plotting of contig properties for these genus bins allowed for an approximate count of the species or strains present within each genus (Figure 3). Within the scatterplots, contigs that form distinct clusters share similar GC content and coverage within the metagenome and can be assumed to originate from the same organism or organisms that are closely related and have similar abundance within the community. The presence of multiple distinct clusters within some genus bins suggests that multiple unique species or strains within that genus were present in the community. In particular, there was a high-abundance *Micromonospora* cluster accompanied by several lower abundance clusters in the thermophilic community (Figure 3A). The *Mycobacterium* bin in the thermophilic community lacked a high abundance cluster on the order observed for *Micromonospora* but did contain multiple lower abundance clusters (Figure 3B). Contigs from these bins had high GC content. *Pseudoxanthomonas* clusters were also predominantly high GC and prominent clusters were observed in both the thermophilic and mesophilic communities (Figure 3C–D). The highest abundance *Pseudoxanthomonas* cluster in each community shared similar GC contents, suggesting they may correspond to the same or similar organisms. *Chryseobacterium* clusters in the mesophilic community had GC contents generally below 50% and a single high abundance cluster was observed (Figure 3E). Although they were not major contributors to the overall dissimilarity between the two communities, notable clusters were observed in several other genus bins. For example, *Niabella* in the thermophilic community contained one highly abundant cluster with large contigs, suggesting well-assembled sequences (Figure 3F). Similarly, *Niastella* and *Chelativorans* in the mesophilic and thermophilic communities, respectively, contained high abundance, well-assembled clusters (Figure 3G–H).

Clusters were screened for ribosomal and non-ribosomal phylogenetic marker COG genes to gauge the completeness of their genomes. The Joint Genome Institute’s list of seventy such marker COG genes was used. These genes are expected to be broadly conserved and contain species-specific sequences. As these conserved marker genes are typically spread out across microbial genomes, a cluster was considered to have captured the majority of an organism’s genome if it contained a complete set of marker COG genes and had a total sequence length comparable to published genomes in the same genus. This analysis revealed varying levels of genome content in each cluster (Table 5). The high abundance *Micromonospora* and *Niastella* clusters in the thermophilic and mesophilic communities, respectively, both contained a complete set of marker COG genes. Additionally, the total sequence length within the *Micromonospora* cluster is similar to other genomes within this genus. These data suggest that metagenome sequenced potentially captured a near complete genome for this particular *Micromonospora* species. Although, no other *Niastella* genomes have been sequenced for comparison, the presence of a complete set of marker COG genes and total sequence length comparable to bacterial genomes suggest most of the genome for this organism may have been captured as well. Similarly, the high abundance mesophilic *Chyseobacterium* and thermophilic *Niabella* clusters have near complete sets of marker genes, suggesting the majority of their genome sequence are represented in their respective clusters. The high abundance *Psuedoxanthomonas* clusters in both the thermophilic and mesophilic communities, as well as the thermophilic *Chelativorans* cluster, were only partially assembled, with marker genes counts indicating that 33–69% of these genomes are represented in the cluster sequences. Furthermore, as most marker COG genes are expected to occur as a single copy per genome, the average gene count across all marker COGs with at least one hit was used as an indicator of how many species or strains were represented within each cluster. For all clusters, the average gene count within all detected marker COGs was less than 1.5, suggesting the presence of only a single species or strain within each cluster and minimal errors in assembly or binning.

**Table 5:** Contig cluster properties for selected clusters (Figure 3) with high abundance or large contigs in thermophilic and mesophilic communities.

Cluster	Community*	Taxonomy	Number of markerCOG genes (outof 70)	Mean count fordetected markerCOG genes	Total sequencelength in cluster(Mb)	Average genome sizein IMG database(Mb)
1	T	*Micromonospora*	70		6.6	6.9
		(*Actinobacteria*)		1.29		
2	T	*Pseudoxanthomonas*	23		1.6	3.4
		(*Proteobacteria*)		1.04		
3	M	*Pseudoxanthomonas*	48		3.3	3.4
		(*Proteobacteria*)		1.38		
4	M	*Chryseobacterium*	68		4.4	5.6
		(*Bacteroidetes*)		1.46		
5	T	*Niabella*	64		3	n/a
		(*Bacteroidetes*)		1.03		
6	M	*Niastella*	70		6.6	n/a
		(*Bacteroidetes*)		1.03		
7	T	*Chelativorans*	38		3.1	4.9
		(*Proteobacteria*)		1.08		

* T, thermophilic community; M, mesophilic community.

### Protein Families in Metagenomes Relevant to Rice Straw Deconstruction

Metagenomes were compared to find protein families overrepresented in the thermophilic community relative to the mesophilic community. For these particular communities, a critical p-value of 4.46e-3 denoted statistical significance. Among the four most overrepresented protein families in the thermophilic enrichment, carbohydrate-binding module family 2 (CBM2) was significantly enriched in the thermophilic community (p<1e-15) with 91 hits (equaling a normalized frequency of 208.4) in the thermophilic community versus 39 (equaling a normalized frequency of 45.9) in the mesophilic community. The second and third most abundant CBMs in the thermophilic community were CBM48 and CBM4/9 with 86 and 34 hits, respectively. In contrast to CBM2, both CBM48 and CBM4/9 were significantly underrepresented in the thermophilic community compared to the mesophilic community (p = 1.2e-3 for CBM48 and p = 1.87e-7 for CBM4/9).

**Figure 4:**
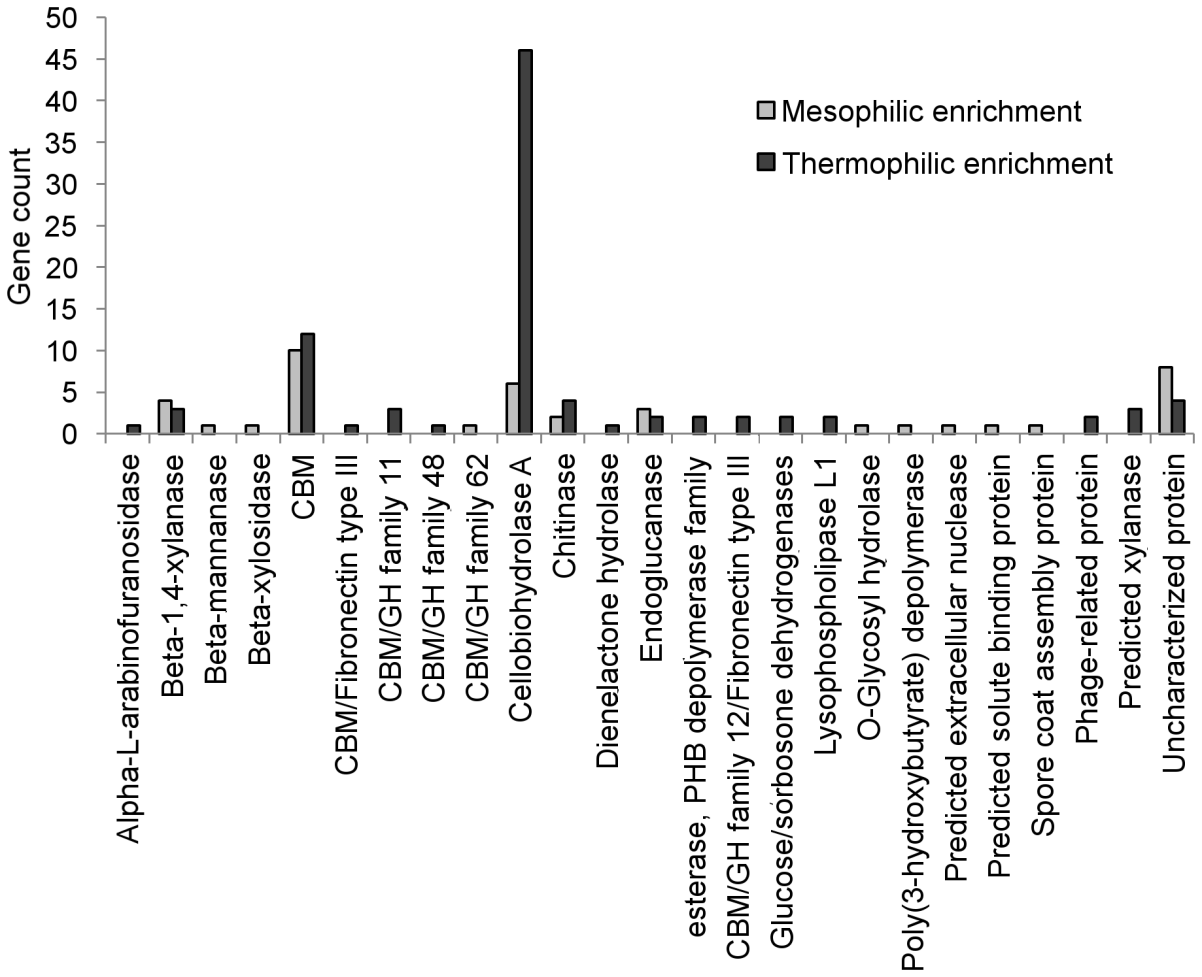
COG classifications of genes containing CBM2 motifs in microbial communities from thermophilic and mesophilic enrichments.

**Figure 5:**
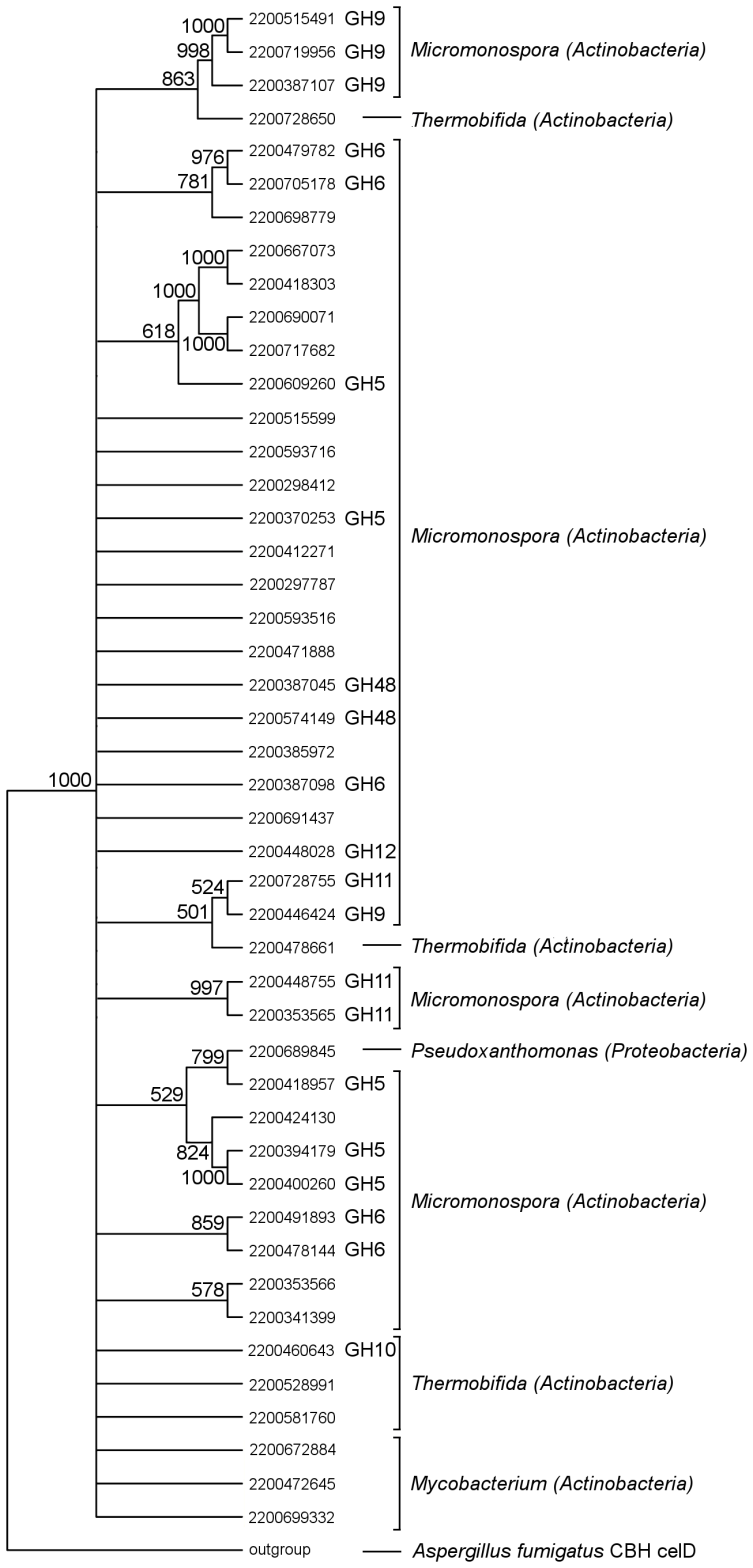
Consensus neighbor-joining tree of CBH-A genes with CBM2 in the thermophilic microbial community. Genes are represented by their IMG gene object ID numbers. For genes that had a glycoside hydrolase (GH) family ascribed to them during annotation, GH family number is indicated next to the gene object ID. Numbers at nodes denote the percentage of trees that support that node out of 1000 bootstrap replicates.

COG classifications for all genes containing CBM2 revealed that overrepresentation of CBM2-containing genes in the thermophilic community stemmed primarily from overrepresentation of cellobiohydrolase A (CBH-A) genes (COG 5297) (Figure 4). Several CBH-A genes exhibited similarity with respect to their glycoside hydrolase (GH) family (Figure 5). Out of 46 genes, 45 were housed on contigs binned to *Actinobacteria*. Of the 45 CBH-A genes binned to *Actinobacteria*, 37 were binned to the genus *Micromonospora,* 5 were binned to *Thermobifida,* and 3 were binned to *Mycobacterium.* Notably, 22 of the 46 CBH-A genes with CBM2 in the thermophilic community mapped back to the high abundance *Micromonospora* cluster (cluster 1 in Figure 3A). Several of the CBH-A gene sequences were fragmented. As a result, while there was enough sequence present to facilitate annotation as a CBH-A and genus binning, the fragmentation prevented meaningful neighbor-joining of these genes. Furthermore, such fragmentation may have also prevented assignment of these sequences to GH families. These fragmented sequences are largely reflected in the similarity tree presented in Figure 5 as genes that only branch with respect to the outgroup.

Abundant and well-assembled clusters were screened for all GH protein families (Table 6). GH hits were compared to the CAZy database [40] to isolate those relevant to lignocellulose deconstruction. Clusters contained a range of GH genes spanning cellulases and hemicellulases. The high abundance thermophilic *Micromonospora* cluster contained endoglucanases and several types of hemicellulases in addition to the overrepresented CBH genes noted previously. Hemicellulases corresponded to GHs active on xylan, mannan, and arabinan. High abundance *Pseudoxanthomonas* clusters in both the mesophilic and thermophilic communities also exhibited a variety of cellulases and hemicellulases. The thermophilic cluster registered fewer GHs, perhaps owing to a less complete assembly than that in the mesophilic community. The high abundance *Chryseobacterium* cluster in the thermophilic community contained mostly hemicellulases. *Bacteroidetes* clusters in the thermophilic and mesophilic communities contained cellulases and hemicellulases. Compared to other clusters, there were more hemicellulases in the GH 43 family present in the *Bacteroidetes* clusters.

**Table 6:** Glycoside hydrolase genes relevant to lignocellulose deconstruction in high-abundance organisms within thermophilic and mesophilic community metagenomes.

			Number of hits within cluster sequence						
Proteinfamily	GHfamily	Dominant types	Cluster 1	Cluster 2	Cluster 3	Cluster 4	Cluster 5	Cluster 6	Cluster 7
pfam00150	5	β-mannosidaseendo-β-1,4-glucanaseendo-β-1,4-mannosidaseendo-β-1,4-xylanaseβ-1,4-cellobiosidaseβ-1,3-mannanasexyloglucan-specificendo-β-1,4-glucanaseexo-β-1,4-glucanase	2	0	1	0	1	5	0
pfam00232	1	β-glucosidase	2	0	0	0	0	1	0
pfam00331	10	endo-1,4-β-xylanaseendo-1,3-β-xylanase	5	0	1	0	2	0	0
pfam00457	11	xylanase	2	0	1	0	0	0	0
pfam00722	16	endo-1,3-β-glucanaseendo-1,3(4)-β-glucanasexyloglucanase	2	0	1	2	0	6	0
pfam00759	9	endoglucanasecellobiohydrolaseβ-glucosidase	2	1	3	0	1	2	0
pfam00933	3	β-glucosidase1,4-β-xylosidaseexo-1,3-1,4-glucanaseα-L-arabinofuranosidase	10	2	4	3	2	3	1
pfam01270	8	celluloseendo-1,4-β-xylanasereducing-end-xylose releasingexo-oligoxylanase	0	1	1	0	0	0	0
pfam01341	6	endoglucanasecellobiohydrolase	4	0	0	0	0	0	0
pfam01670	12	endoglucanasexyloglucan hydrolaseβ-1,3-1,4-glucanase	1	0	0	0	0	0	0
pfam01915	3C	β-glucosidase1,4-β-xylosidaseexo-1,3-1,4-glucanaseα-L-arabinofuranosidase	5	1	5	2	1	3	0
pfam02011	48	reducing end-actingcellobiohydrolaseendo-β-1,4-glucanase	1	0	0	0	0	0	0
pfam02156	26	β-mannanaseβ-1,3-xylanase	0	0	0	0	0	3	0
pfam03648	67N	α-glucuronidasexylan α-1,2-glucuronidase	1	0	1	0	1	3	0
pfam03664	62	α-L-arabinofuranosidase	1	0	0	0	0	0	0
pfam04616	43	β-xylosidaseα-L-arabinofuranosidasearabinanasexylanase	3	0	4	0	11	11	0
pfam07477	67C	α-glucuronidasexylan α-1,2-glucuronidase	1	0	2	0	1	1	0
pfam07488	67M	α-glucuronidasexylan α-1,2-glucuronidase	1	0	2	0	1	1	0

## Discussion

### Microbial Activity and Extracted Endoglucanase and Xylanase Activities

Thermophilic incubations on rice straw yielded higher microbial activity and extracted enzyme activity levels than mesophilic incubations. The higher activity at 55°C is consistent with other observations of plant biomass decomposition. For instance, food waste and green waste composts decomposed two times faster at 45°C compared to 35°C [41]. The higher decomposition and enzyme activity levels observed at 55°C support the use of thermophilic environments for discovery of organisms and enzymes for biomass deconstruction.

Enzyme extraction from incubated rice straw increased with increasing concentrations of ethylene glycol in the extraction buffer. The results suggest secreted enzymes have strong hydrophobic interactions with rice straw polysaccharides. Such interactions have been identified for carbohydrate binding modules associated with cellulases [42], [43]. Similar effects of ethylene glycol were observed for the extraction of xylanase and endoglucanase from corn stover and switchgrass incubated under high-solids thermophilic conditions [15]. These observations indicate thermophilic, high-solids biomass deconstruction systems favor organisms that secrete enzymes with strong hydrophobic plant cell wall interactions and that enzyme binding plays an important role in these systems.

### Microbial Community Composition

The largest contributor to dissimilarity between the thermophilic and mesophilic communities was *Micromonospora*. Certain species within *Micromonospora* have been characterized as cellulose degraders and thermophiles [44]–[47]. Enrichment of *Micromonospora* under thermophilic conditions in this study is consistent with these prior observations. Moreover, several *Micromonospora* species have been shown to be capable of deconstructing rice straw in liquid culture and compost systems [48]–[50]. These data support the possibility that enrichment of *Micromonospora* species in this study under thermophilic conditions corresponds to these species taking a more active role in rice straw deconstruction compared to mesophilic conditions. Both mesophilic and thermophilic communities contained high-abundance genera within *Proteobacteria* and *Bacteroidetes* phyla. Prevalence of *Pseudoxanthomonas* in both enrichments is in agreement with previous studies that have found *Pseudoxanthomonas* species to be major components of bacterial consortia with high cellulolytic activity under mesophilic and thermophilic conditions [51]–[53]. In contrast, other *Proteobacteria* genera like *Chelativorans*, which was detected in high abundance in the thermophilic community, are not well studied with respect to cellulolytic activity and have not been reported as lignocellulose degraders.

*Bacteroidetes* genera found in the thermophilic and mesophilic communities have previously been isolated from arboreal and greenhouse soils. Notably, *Niabella* species isolated from soils grew only under mesophilic conditions [54]–[56]. Tolerance to temperatures as high as 55°C, as observed here, has not been reported previously for this genus. Moreover, no *Niabella* isolates to date have exhibited the ability to hydrolyze carboxymethylcellulose [54], [55], [57]. Alternately, several *Chryseobacterium* species have been detected in cellulose-degrading gut communities [58], [59]. Likewise, species within the genus *Niastella* have been isolated from arboreal soils and several have exhibited the ability to hydrolyze carboxymethylcellulose [60]. *Niastella* isolates are typically mesophiles [56], [60], which may explain the decrease in *Niastella* abundance seen under thermophilic conditions in this study. The data presented here demonstrate that temperature may significantly impact the community members and enzymes responsible for lignocellulose degradation, a vital consideration when using metagenomics to discover lignocellulolytic enzymes for biofuel production.

### Community Metagenomes and Determinants of Lignocellulolytic Activity

Lignocellulolytic enzymes detected in high-abundance and well-assembled metagenome contig clusters compliment organism abundance data to help elucidate each species’ potential role in rice straw deconstruction. Comparison of genes containing protein family domains relevant to lignocellulose deconstruction in each metagenome provides an avenue for identifying promising enzymes for biofuels applications. In this study, the thermophilic metagenome was screened for protein families that were represented in significantly greater quantities compared to the mesophilic metagenome. Such overrepresented protein families may indicate specific genes that confer a selective advantage to their host organism under thermophilic conditions. Moreover, overrepresented genes encoding lignocellulolytic enzymes present targets for further investigation, as they may represent thermotolerant enzymes that maintain activity under high-solids conditions similar to those necessary for biofuel production. In this research, one such deconstruction-relevant protein family, carbohydrate-binding module family 2, was significantly overrepresented in the thermophilic community. If cellulases containing CBM2 do confer an advantage to the *Actinobacteria* that produce them during high-solids culture on rice straw, it may be due to increased activity of these enzymes under thermophilic conditions. A variety of cellulose-binding CBMs exist in nature with varying affinities to different plant cell walls, potentially exploiting various structural changes that result from cellulose interactions with other cell wall components [61]. Cellulases with CBM2 may be better able to bind cellulose within the unique structure of rice straw cell walls. Additionally, binding of cellulases with CBM2 to cellulose may be more stable compared to other CBMs under thermophilic conditions or the structure of CBM2 itself may be more thermostable. Further characterization of CBM2 is needed to assess these possibilities. Since most CBM2-containing enzymes in the thermophilic community were cellobiohydrolases derived from *Actinobacteria* (high-abundance *Micromonospora* in particular), these CBHs also warrant further investigation as thermophilic enzymes for high-solids rice straw deconstruction.

Although they were not a major contributor to overall community dissimilarity nor did they contain significantly overrepresented protein families, *Niabella* bacteria present in high abundance in the thermophilic community did contain more family 43 GHs compared to other deconstruction-relevant GHs. Analysis of marker COG genes suggests that most of this *Niabella* species’ genome is represented in the metagenome sequence and, as a result, it can be reasonably assumed that the observed asymmetry in GHs is truly representative of this organism’s genome. As family 43 GHs are active on hemicellulose, *Niabella* may primarily utilize hemicellulose during rice straw decomposition. Previous characterization of *Niabella* species have only examined activity on cellulose and have neglected hemicellulose polysaccharides [54], [55], [57]. As none of these previously studied isolates were active on cellulose, the observed abundance of hemicellulase genes in this work provides motivation to investigate hemicellulolytic activity in this genus and to determine if *Niabella* family 43 GHs represent enzymes for hemicellulose deconstruction in high-solids environments.

This work demonstrates the usefulness of the metagenomic approach for identifying genes of interest in microbial communities enriched to select for organisms capable of deconstructing rice straw under industrially relevant conditions. This technique can presumably be applied to other microbial community systems to identify target genes with industrially-applicable capabilities. The metagenomic approach gauges the abundance of specific organisms and provides insight into their potential capabilities by revealing the genes they possess. However, it must be noted that metagenomics provides no indication of whether organisms actually express these genes. As a result, additional metatranscriptomic and metaproteomic analyses are required to ultimately confirm their activity within enrichment cultures.
